# Risk factors associated with dystocia in swine

**DOI:** 10.14202/vetworld.2021.1835-1839

**Published:** 2021-07-16

**Authors:** Nguyen Hoai Nam, Peerapol Sukon

**Affiliations:** 1 Faculty of Veterinary Medicine, Vietnam National University of Agriculture, Trauqui, Gialam, Hanoi, Vietnam; 2 Faculty of Veterinary Medicine, Khon Kaen University, 123 Moo 16 Mittraphap Road, Nai-Muang, Muang District, Khon Kaen 40002, Thailand; 3 Research Group for Animal Health Technology, Khon Kaen University, 123 Moo 16 Mittraphap Road, Nai-Muang, Muang District, Khon Kaen 40002, Thailand

**Keywords:** birth order, crown rump length, dystocia, piglet, swine

## Abstract

**Background and Aim:**

Dystocia in swine can increase the stillbirth rate; however, its importance in pig-breeding systems has been underestimated. Until now, few studies have investigated dystocia and associated risk factors. Therefore, in this study, we aimed to evaluate the effects of various risk factors on dystocia in swine.

**Materials and Methods:**

Out of 5,557 piglets, we included 4,997 piglets in risk analysis study. The dependent variable was dystocia, which was recorded when a birth interval exceeded 45 min or when obstetric assistance was applied. The independent variables were parity, gestation length, litter size, birth order, gender, presence of a dead piglet, birthweight, crown rump length, body mass index, ponderal index, and oxytocin use. We used generalized linear mixed models to examine the associations between potential risk factors and dystocia at the piglet level.

**Results:**

A total of 6% of the piglets were born with a dystocia event, and 47.2% of the farrowing experienced at least one event. Dead piglets and piglets with a crown rump length of >31 cm were associated with an increased dystocia rate. This rate decreased from birth order 2 to 7, stabilized to 11, and then increased till the end of the fetal expulsion process.

**Conclusion:**

Dystocia is common in swine. Therefore, this condition should be carefully addressed by veterinarians and farrowing house personnel so that its adverse effect on welfare and productivity of sows and survivability of piglets can be reduced. Further studies investigating dystocia status and risk factors in different swine farrowing systems should be undertaken to provide more knowledge about this neglected condition.

## Introduction

To date, few studies have described dystocia in swine. Cowart [1] has defined dystocia as failure in the delivery of piglets within 2 h from the beginning of the farrowing or failure in the delivery of a successive piglet within 1 h. More recently, Ward *et al*. [2] have reported that a dystocia event in sows occurs when a birth interval exceeds 45 min. In swine, perinatal survival is considered to be much more important than dystocia, resulting in little attention paid to this farrowing abnormality [3].

In the literature, several causes of dystocia in swine have been discussed, including uterine inertia, obstruction of the birth canal, fetal malposition, and fetomaternal disproportion [4]. A gestation length exceeding 116 days may also be a sign of dystocia [1]. In a review, Parkinson *et al*. [3] have reported that 0.25-3.0% of all farrowing have experienced dystocia. Definition of dystocia based on birth interval [2] may significantly elevate the dystocia rate in swine. Studies have shown that birth interval is associated with birthweight, stillbirth, piglet presentation, birth order, and litter size [5-7]. Hence, these factors may also be associated with dystocia. More importantly, the categorization of prolonged birth interval as part of dystocia will establish an easy guide for when to apply obstetric intervention to assist sows and piglets [2,8,9], thereby reducing the stillbirth rate.

The current literature lacks studies investigating the risk factors for dystocia in swine. Therefore, in this study, we aimed to evaluate the effects of various risk factors on dystocia in swine.

## Materials and Methods

### Ethical approval

No animal samples were used in this study, and the observation and measurement were done in a humane way to avoid any stress to investigated animals.

### Study period and location

This study was conducted from February to November 2019 in six swine farms in the North of Vietnam.

### Animals

We recorded information on 5,557 piglets born from 387 Landrace×Yorkshire crossbred sows. Pregnant sows received 1.8-4.0 kg of industrialized feed per day and water from a bite nipple system *ad libitum*. Farrowing sows were allocated into individual 1.8 m×2.2 m farrowing crates with a centered sow area of 0.6-0.7 m×2.2 m. All sows were vaccinated against classical swine fever, foot-and-mouth disease, porcine circovirus, porcine respiratory and reproductive syndrome, and Aujeszky’s disease. Sows were supervised throughout the fetal expulsion stage by at least one veterinarian.

### Data collection and definition

In our data collection, we recorded parity number, gestation length, litter size, birth interval, birth order, gender, birthweight, crown rump length, and use of oxytocin. Litter size was the sum of the numbers of piglets born alive, stillborn, and mummified. Birth interval was the interval between the births of two successive piglets; therefore, the first piglets of all litters did not have a birth interval. We humanely measured birthweight and crown rump length immediately after the piglets were cleaned with cloth or dried with hygroscopic flour.

We calculated body mass index (BMI) and ponderal index as birthweight (kg) divided by squared crown rump length (m^2^) and cubed crown rump length (m^3^), respectively. We defined stillborn piglets as those that died before expulsion and had no signs of decay [10]. Dead piglets included those stillborn or mummified. We recorded birth orders at which oxytocin (20 UI/dose) was used.

We recorded an event as dystocia when a birth interval exceeded 45 min [2] or when obstetric assistance was applied [8]. We defined the dystocia rate at the piglet level as the total number of dystocia events divided by the total number of piglets born and the rate at the sow level as the number of farrowing with at least one dystocia event divided by the total number of farrowing.

### Statistical analysis

We excluded piglets without full information thereby leaving 4,997 piglets used in risk analysis. We used generalized linear mixed models (GLMMs) to identify potential risk factors for dystocia at the piglet level. Independent variables were gender, use of oxytocin, piglets born dead, parity, litter size, gestation length, birth order, birthweight, crown rump length, BMI, and ponderal index. In all models, sows nested in different farms were fitted as random factors to consider the litter differences and any variation in farms [11].

GLMMs started with univariate analysis to determine the most significant risk factor for dystocia, which was then combined with other factors significant at p≤0.1 and were analyzed with different multivariate GLMMs to establish the final model that best explained the dystocia variation. We then partitioned the continuous independent variables selected in the final model into categorical variables to examine the effect of these factors on dystocia at different ranges. We used the Hosmer–Lemeshow goodness-of-fit test to examine if the observed outcome matched the expected outcome. We conducted model building in RStudio Desktop 1.3.1093 (RStudio Team: Integrated Development for R, Boston, MA, USA). We set p≤0.05 as the level of statistical significance in the final model.

## Results

The dystocia rate at the piglet level and stillbirth and mummification rates were 6.0%, 8.7%, and 1.6%, respectively. Nearly half of the farrowings (47.2%) experienced at least one dystocia event. The incidence of oxytocin use at farrowing level was 45.0%. The birth interval and farrowing duration were 17.2±33.9 min and 224.2±143.6 min, respectively. Birthweight and crown rump length were 1.4±0.4 kg and 27.4±3.1 cm, respectively.

Univariate analysis showed that, apart from male piglets (odds ratio [OR]=1.11; 95% confidence interval [CI]=0.87–1.41), birthweight (OR=1.30; 95% CI=0.92–1.84), use of oxytocin (OR=0.91; 95% CI=0.56–1.47), parity (OR=1.00; 95% CI=0.95–1.07), gestation length (OR=0.99; 95% CI=0.91–1.07), and BMI (OR=0.97; 95% CI=0.94–1.01), other risk factors including dead piglets (OR=2.00; 95% CI=1.24–3.22), litter size (OR=0.94; 95% CI=0.90–0.98), birth order (OR=0.96; 95% CI=0.93–0.99), crown rump length (OR=1.07; 95% CI=1.03–1.12), and ponderal index (OR=0.99; 95% CI=0.99–0.99) were associated with dystocia.

The final GLMM that best explained the variation of dystocia (18.0%) selected dead piglets, birth order, and crown rump length as the most significant risk factors (Table-1). The presence of dead piglets and a crown rump length of >31 cm increased the risk of dystocia. Piglets with birth order 7–11 had the lowest odds of dystocia, whereas those with birth order 2 had the highest odds. The odds decreased gradually from birth order 2 to 7, stabilized to 11 and then increased till the end of the fetal expulsion process.

**Table-1:** Multivariate generalized linear mixed models for the association between potential risk factors and dystocia in 4997 piglets born from 387 sows in six herds in the North of Vietnam.

Covariates	Incidence of dystocia	OR; 95%CI	p-value
Born alive	5.5 (252/4564)	1	
Born dead	11.5 (50/433)	2.17; 1.53–3.07	<0.001
Birth order=2	13.8 (53/384)	4.60; 3.14–6.71	<0.001
Birth order=3	8.9 (34/384)	2.60; 1.70–3.96	<0.001
Birth order=4–6	5.7 (64/1131)	1.48; 1.05–2.09	0.003
Birth order=7–11	3.8 (67/1752)	1	
Birth order=12–16	5.8 (65/1123)	1.62; 1.15–2.28	0.006
Birth order=17–23	8.5 (19/223)	2.29; 1.32–3.95	0.003
Crown rump length=22–31 cm	5.5 (240/4359)	1	
Crown rump length <22 cm	7.1 (9/126)	1.08; 0.56–1.93	0.81
Crown rump length >31 cm	10.4 (53/512)	2.02; 1.40–2.90	<0.001

OR=Odds ratio, CI=Confidence interval, P=Probability level. The final multivariate model with the overall significance <0.001 selected dead piglet, birth order, and crown rump length as the most significant factors for dystocia in swine. Hosmer–Lemeshow goodness-of-fit test showed a good fit between expected and observed outcomes (p-value=0.611). The marginal and conditional R-square were 0.065 and 0.180, respectively.

## Discussion

In this study, we first report the dystocia rate at the piglet level (6.0%, 308/5137 piglets), which we defined as the percentage of piglets born with a dystocia event. For dystocia rate calculation we excluded piglets that did not have information on birth interval. Therefore, 5137 piglets with available birth intervals were used for this purpose. The published literature has defined the dystocia rate (i.e., the percentage of farrowing with at least one dystocia event) in swine as ranging from 0.25% to 3.0% [1,3,4]. By this definition, the dystocia rate at the sow level is strikingly high in our study, up to 35.7–47.2% (138–183/387 sows), depending on the birth interval cut-off of 60 min [1] or 45 min [2].

An explanation of the differences in dystocia rates between the current study and previous studies is particularly challenging. First, a detailed definition of dystocia cannot be retrieved from previous research discussing dystocia rate [3,4]. Second, even in research in which the definition of dystocia is mentioned [1], the gap between the results of this study and the others cannot be bridged. Third, with the increase in litter size in recent decades [12] and due to the negative association between litter size and birth interval [13], the dystocia rate in the current study should have decreased as compared to previous results.

Our study indicates that birth order is a significant factor for dystocia. The effect pattern of birth order on dystocia in the current study is similar to that seen in the previous studies [6,14], which have found that birth interval decreased to 40% [6] or to 60–74% [14] of the relative birth order (i.e., birth order×100/litter size) and then increased toward the end of farrowing. In this study, the dystocia rate also decreased to 50–60% of the relative birth order and then increased. The dystocia rates of the second (13.8%) and the last (11.5%) piglets in this study were higher in comparison to the dystocia rates of the other piglets; this corroborates the finding by Motsi *et al*. [6] that the birth interval of the first two and the last two piglets was longer than the average birth interval.

The preparation of the uterus for farrowing and uterine fatigue at late farrowing is possible explanation for the quadratic effect of birth order on dystocia demonstrated in this study. Plasma oxytocin concentration of crated sows increases after the birth of the first piglets and during the 1^st^ h of delivery and then decreases when the farrowing goes beyond 1 h [15], which can explain why the dystocia rate gradually reduced from the birth of the 2^nd^ piglets to the birth of the 7^th^–11^th^ piglets in this study. It is possible that the increase in dystocia rate at the second half of farrowing is secondary to the decrease in plasma oxytocin concentration [15] or the fatigue of the uterus [6,16].

The positive association between dead piglets and dystocia in this study confirms previous results showing increased rates of stillbirth during a prolonged birth interval [5,13,17]. It is difficult to determine the cause-and-effect relationship between dead piglets and increased dystocia. However, a mutual association seems to exist between these two factors. On the one hand, dystocia increases the time periods that piglets can experience hypoxia and asphyxia; therefore, they are more likely to be born dead. On the other hand, dead piglets cannot actively move through the reproductive tract [18], subsequently increasing the dystocia rate.

In this study, the crown rump length of the piglets was more significant than their birthweight, BMI, and ponderal index values in the explanation of dystocia in the sows. The increased dystocia rate in the piglets with a crown rump length of >31 cm may be due to their larger size. In comparison to the lighter piglets, the heavier piglets had thicker placental membranes; hence, they may require a longer time to rupture this membrane [14]. In addition, the larger piglets created higher friction on the walls of the reproductive canal [6] and so required a longer duration to travel through. Moreover, the increased dystocia rate in piglets with a crown rump length of >31 cm can be due to the fact that these were more likely to be born dead than were the piglets with a crown rump length of 22-31 cm (i.e., 12.5% [53/423] *vs*. 8.1% [352/4365]).

The negative association between litter size and dystocia corroborates previous findings that increased litter size can decrease birth interval [13]. The relationship between these two factors can be explained by the negative association between litter size and crown rump length (r=−0.14; p<0.001) and birthweight (r=−0.176; p<0.001) in this study.

The current study did not find any effects of the use of oxytocin, parity, and gestation length on dystocia in swine. The non-significant effects of oxytocin may have been caused by a reduction in endogenous oxytocin release at the second half of farrowing after a rise in the early fetal expulsion stage [15]. The use of oxytocin at high birth order (8.4±4.6) in this study acted as a mild stimulation to the fatigued uterus at the late stage of expulsion [19] and therefore did not cause any difference in dystocia rate pre- and post-injection. The non-significant association between parity and dystocia in our study may be caused partly by the selection effect in which older sows with bad reproductive performance and maternal abilities were removed from the herds. The fact that gestation length did not have any association with birthweight and crown rump length can be a reason for its non-significant effect on dystocia.

## Conclusion

Unlike previous research indicating that dystocia in swine is a minor issue [3], our study demonstrated that dystocia events are common in this species and are experienced in a large proportion of farrowing. Low and high birth order, a piglet crown rump length of >31 cm, and piglets born dead are most significantly associated with increased dystocia in swine. This knowledge is helpful for veterinarians and farrowing house personnel who deal with dystocia in order to reduce its adverse effect on welfare and productivity of sows and survivability of piglets.

## Authors’ Contributions

NHN: Collected the data. NHN and PS: Conceived and designed the study, analyzed data, interpreted results, and wrote the manuscript. Both authors read and approved the final manuscript.
